# Overview of the Japan Children’s Study 2004–2009; Cohort Study of Early Childhood Development

**DOI:** 10.2188/jea.JE20100018

**Published:** 2010-03-05

**Authors:** Zentaro Yamagata, Tadahiko Maeda, Tokie Anme, Norihiro Sadato, Japan Children’s Study Group

**Affiliations:** 1 Research Institute of Science and Technology for Society, Japan Science and Technology Agency, Tokyo, Japan; 2 Department of Health Sciences, Interdisciplinary Graduate School of Medicine and Engineering, University of Yamanashi, Chuo, Yamanashi, Japan; 3 The Institute of Statistical Mathematics, Research Organization of Information and Systems, Tachikawa, Tokyo, Japan; 4 International Community Care and Lifespan Development, Graduate School of Comprehensive Human Sciences, University of Tsukuba, Tsukuba, Ibaraki, Japan; 5 Department of Cerebral Research, National institute for Physiological Sciences, Okazaki, Aichi, Japan

**Keywords:** sociability, development, cohort study, neuroscience

## Abstract

**Background:**

There are still a lot of unknown aspects about the childhood development of sociability which are based on neuroscientific basis. Purpose of the Japan Children’s Study (JCS) was to verify the normal process of child development of sociability; the trajectory and factors related development of sociability, and to collect findings and integrate the knowledge to make the plan of long-term and large scale cohort study.

**Methods:**

A child cohort study underway in Japan since 2005. There are the cohort study including a infant cohort study at age of 4 months to 30 months and a preschool cohort study at age of 5 years old to 8 years old. Questionnaires, direct observation of children and cognitive testing were performed.

**Results:**

In infant cohort study, 465 infants were recruited at 4 months and 367 children were followed up to 30 months, follow up rate was 78.9% and in the preschool cohort study, total 192 children (112 at 2005 and 80 at 2007) at age of 5 years old and 169 followed up to 6 years (follow up rate was 88.0%), and 79 children were followed up to 8 years old (follow up rate was 70.5%) old. Several new measurements to evaluate child sociability were developed. Some factors related to development of child sociability were found for example the ‘praise’ was related to child sociability in cohort study based on neuroscience findings.

**Conclusions:**

Though the trajectory of child sociability development were not clarified, some significant factors related to development of sociability, and the basic findings to conduct a long-term and large scale cohort study were provided.

## INTRODUCTION

Recently, children have been difficult to acquire their sociability and the children with behavioral symptom of a lack of sociability, such as a lack of self-control, absenteeism, developmental disorder, delinquency, withdrawal, concerns about childcare, and child abuse have been difficult to live in our society.^1^^–^^3^ This situation also has been a social problem. Sociability is the ability to build human relationships and generally this ability is known as “the ability to get along with others.” Therefore, this encourages making the interpersonal environment.^4^

However, there are still a lot of unknown aspects about the childhood development of sociability which are based on neuroscientific basis. Particularly, some specific environmental factors which influence the development of the psyche of children mainly in the initial phase of the development of sociability during infancy are still largely obscure. There were some reports that the childhood development of sociability strongly depended on their individual factors, such as their character, congenital growth and subsequent development.^5^^,^^6^

On the other hand, the fostering behavior of the people who are raising the child and mother-child relationships could modify the character of the child from the view of family and social environment. Therefore, some researcher emphasized that environmental factors might be more important than the individual factors.^5^ Moreover, it is necessary to clarify several questions related the process of childhood development of sociability which are influenced by various biological and psychosocial factors.

Purpose of The Japan Children’s Study (JCS) was to verify the normal process of childhood development of sociability using a questionnaire, direct observation of children which was conducted by researchers or developmental psychologists and pediatricians, cognition tests and functional brain imaging. This cohort study also aimed to integrate the knowledge of researchers who are in various field to make the plan of long-term and large scale cohort study. Moreover, this study was the first study about the childhood development of sociability in Japan.

## PURPOSES

The purposes of the JCS were (1) to clarify the development of sociability in infants (0–3 years old), and in children (5–8 years old) and the proposition of a hypothetical pattern of childhood development, (2) to clarify the factors which influence the development of sociability, and (3) to collect and integrate the knowledge which were necessary to carry out a large-scale cohort studies as the basis of future long-term research.

In addition, this study also aimed to develop new measurements and methods to estimate sociability as well as new analytical method to process the data and to discuss about implementation of neuroethics for conducting the cohort study and applying the results of this study to public. Moreover, this study could suggest the procedures to implement the system of cooperation between school teachers and researchers as well as to introduce neuroscientific findings to general society.

## STUDY DESIGN AND STUDY GROUPS

The JCS consisted of two cohorts started in 2005 and followed up for 3 years. One of these was an infant cohort study from 4 months old to be followed up to ages of 9, 18, and 30 months in Osaka and Mie prefecture. The other was a preschool cohort study started from 5 years old to 8 years old in Tottori prefecture.

The JCS was an interdisciplinary study and major of the researcher of this study were composed of neuroscience, pediatrics, developmental psychology, pedagogy, epidemiology, statistics and several others.

This study was conducted on the basis of the research system which consisted of the cohort study group and the development neuroscience study groups. The cohort study group conducted their cohort studies in Osaka, Mie, and Tottori prefecture. Their studies consisted of observation of children and their parents and collection of the data.

The development neuroscience group were composed of some subgroups; Neurobehavioral research group, Developmental psychology group, Cognitive experiment group, Neuroimaging group, Measure development group, Sleep research team, Behavioral measurement group, Information and statistics group and Neuroethics research group.

**Figure 1.**
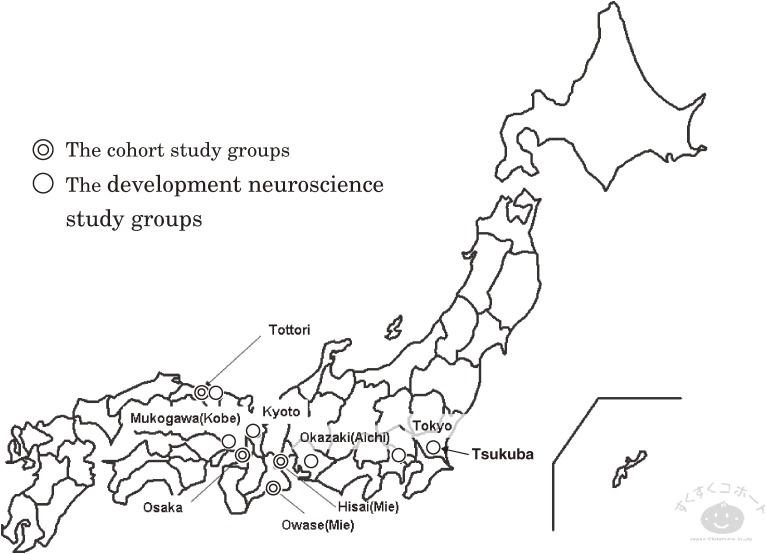
The Locations of the Japan Children’s Study Group

These groups conducted the studies to develop new measurements to estimate sociability and analytical method and to research implementation of neuroethics (Figure 1).

## PARTICIPANTS

Participants, children and their mothers, were recruited at the sites of each cohort study groups in 2005. In Osaka, there were two kinds of participants. One of them was the children who recruited at Miyakojima Health Center when they visited this center for 3 months infant health check up. The other was the children who were born in hospitals at Miyakojima ward and they recruited when they visited these hospitals for 1 month infant health check up. In Mie, recruitment was conducted in two hospitals and one clinic for children who were born these hospitals and clinic when they visited 1 month health checkup. In preschool cohort study, the children who are resident in Tottori city and became 5 years old in 2005 and 2007 were recruited in health checkups.

## MEASUREMENTS

Measurements which were used in this study were listed in Table 1.

**Table 1.** Domains, scales and items included in the questionnaire at each assessment occasion

Target of assessment/ ​ Item Domains	Scale names and/or ​ type of items	Number of Items or Scales on each assessment occasion			

		4 months (Baseline)	9 months	18 months	30 months
I. Parental and Family Factors					
Perinatal Information (mother and baby)	—	11 items	—	—	—
Family Structure	—	(Family membership checklists)		(2 items)	(2 items)
Socio Economic Status	—	4 items	4 items	(3 items)	(3 items)
Caregivers characteristics, health status	—	18 items	18 items	14 items	14 items
Usage of child care facilities	—	—	—	3 items	3 items
Family Functions	family APGAR	1 scale (5 items)	1 scale (5 items)	—	—
Maternal parenting stress	(Original Scale)	1 scale (10 items)	1 scale (10 items)	1 item	1 item
Paternal parenting stress	(Original Scale)	1 scale (10 items)	1 scale (10 items)	—	—
Caregiver's stressful life event	—	—	—	1 item	1 item
Maternal physical health status	—	1 item	1 item	2 items	2 items
Maternal mental health A	GHQ12	1 scale (12 items)	1 scale (12 items)	—	—
Maternal mental health B	(Original Items)	—	—	4 items	4 items
Paternal cooperation	—	3 items	3 items	—	—
Parental and Envioronmental Stimulation A	(Original Items)	—	5 items	—	—
Parental and Envioronmental Stimulation B	EESS	—	—	13 items	13 items
Maternal value about childrearing	(Original Scales)	1 scale (8 items)	1 scale (8 items)	—	—
Paternal value about childrearing	(Original Scales)	1 scale (8 items)	1 scale (8 items)	—	—
Mother's attitude toward childrearing	(Original Scales)	6 scales (25 items)	6 scales (25 items)	—	—
Father's attitude towaer childrearing	(Original Scales)	6 scales (25 items)	6 scales (25 items)	—	—
Potential risk for child abuse	(Screening ​ questionnaire)	—	—	15 items	15 items

II. Child Factors					
Child health status	—	—	—	2 items	2 items
Child's vaccination status	—	—	—	1 item	1 item
Sleep and life habits	—	9 items	10 items	3 items	3 items
Child temparament	(Ogura et al. 2007)	6 scales (36 items)	7 scales (42 items)	4 scales (18 items)	4 scales (18 items)
Overall developmental assessment	KIDS-A and KIDS-B	KIDS-A (6 scales)	KIDS-A (6 scales)	KIDS-B (9 scales)	KIDS-B (9 scales)
Early symptoms of autistic behavior	—	—	—	15 items Checklist	—
Problem Behavior	Strengths and Difficulties ​ Questionnaire	—	—	—	5 scales (25 items)

Notes to selected scales (References).					
Family APGAR	Smilkstein G. (1978). The Family APGAR; a proposal for a family function test and its use by physicians.				
	J. Fam. Pract. 6: 1231–1239.				
EESS (Evaluation of Environmental ​ Stimulation -Short version)	Anme, T. (1996). Evaluation for Child Care Environment. Kawasima Publication, Tokyo, Japan (in Japanese).				
	Anme, T. and Segal, U. A. (2004): Implications for the development of children in over 11 hours of centre-based care. Child Care Health Dev 30, 345–52.				
KIDS: Kinder Infant Development Scale ​ (Type A, Type B)	Miyake K, Ohmura M, Takashima M, Yamauchi S, Hashimoto K. (1989). Kinder infant development scale. Manual: Hattatsukagaku Kenkyu Kyoiku Center, Tokyo (in Japanese). 1989 (in Japanese)				
SDQ (Strengths and Difficulties ​ Questionnaire)	Goodman, R. A. Goodman R (1997) The Strengths and Difficulties Questionnaire: A Research Note. Journal of Child Psychology and Psychiatry, 38, 581–586.				
Autistic Behavior Symptoms	lshii, T. & Takahashi, O. (1983). The epidemiology of autistic children in Toyota, Japan: Prevalence. Japanese Journal of Child and Adolescent Psychiatry, 24, 311–321.				
Stressful Life Event	Shiokawa, H. (2007) Development of the Life Event Questionnaire for Parents: Its use and reliability data. Jichi Medical University Journal, 30, 165–172. (in Japanese with English abstract)				
Child Temparament (Original Scales ​ and Items)	Ogura T, Itakura S, Egami S, Kutuski A, Kubo K. Development of Social Cognition in infancy (4): Influence of temperament. Preceedings of the 70th Conference of the Japanese Psychological Association. Kyuushuu univ, Fukuoka, 2006: 1173 (in Japanese).				

To measure and estimate of exposures and outcomes, some scales, such as the Kinder Infant Development (KIDS),^7^ Family APGAR,^8^ EESS (Evaluation of Environmental Stimulation -Short version),^9^^,^^10^ SDQ (Strengths and Difficulties Questionnaire),^11^ Autistic Behavior Symptoms,^12^ Stressful Life Event,^13^ Child Temparament (Original Scales and Items).^14^ Direct observations using neurobehavioral observation batteries by pediatricians which was developed in this study,^15^ Still Face Experiment,^16^ and cognitive testing using personal computer programs like a children’s preference towards the social stimuli (face, gaze, socially causal movements and biological motion)^17^ were conducted. The part of participants in preschool cohort study participated in the brain fMRI study.^18^ In addition, mothers completed a questionnaire for parents (health status, life habits, socio-economic status, discipline policy and so on).

Almost all measurements were used for all participants at each age in the infant cohort study and in preschool cohort study.

Data from neurobehavioral observations and cognitive testing were recorded on video tapes and scored according to the psychologists trained of neurobehavioral observations.

## STATISTICAL ANALYSIS

The basic approach toward the purposes was to analyze the mechanism of the association between various factors and “outcome” variables, such as childhood development, from the statistical view. Similarly, the interests of the development cohort study were to figure out the changes of these factors on each stage of development using the multi-wage longitudinal data. Therefore, linear regression model and generalized linear model were basically conducted in this study.

## ETHICS

This study protocol was approved by the Ethical Review Committee of Research Institute of Science and Technology for Society, Japan Science and Technology Agency and the Ethical Review Committee of each institutes of JCS research groups, based on the Guidelines Concerning Epidemiological Research of Ministry of Education, Culture, Sports, Science and Technology and Ministry of Health Labour and Welfare in Japan.

## MAIN RESULTS

**Figure 2.**
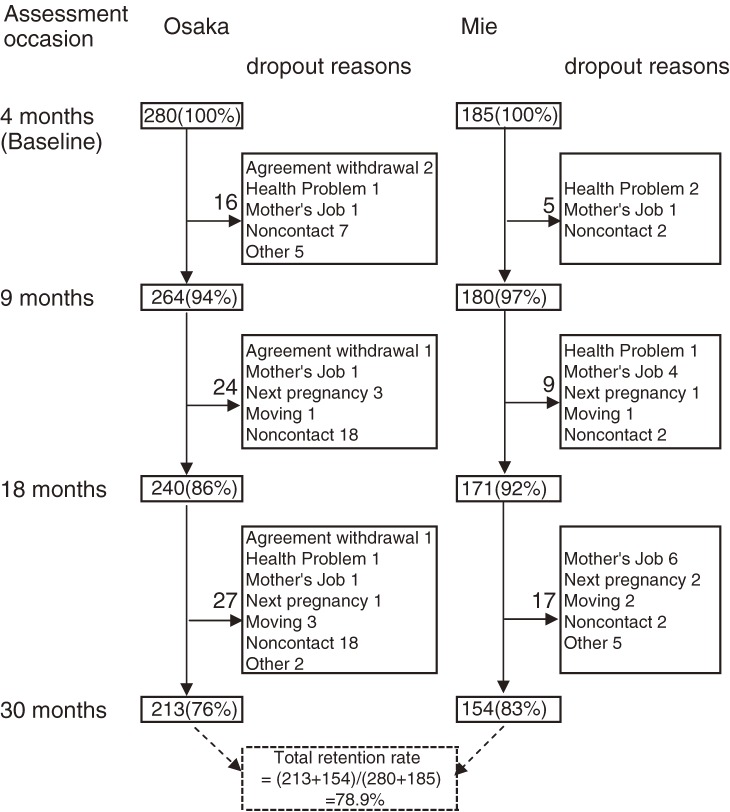
Retention rate until 30 months assessment and reasons for dropouts in each site

The number of participants and follow up rate indicated in Figure 2. In infant cohort study, 465 infants were recruited at 4 months and 367 children were followed up to 30 months, follow up rate was 78.9% and in the preschool cohort study, total 192 children (112 at 2005 and 80 at 2007) at age of 5 years old and 169 followed up to 6 years (follow up rate was 88.0%), and 79 children were followed up to 8 years old (follow up rate was 70.5%) old.

First, regarding the development of sociability in infant, the evaluation of the sign of the development would be possible and this sign would show some possibility to predict later sociability in social cognition when there were a transaction between the person who brought up a child and human sociability.

Development of the examination of the brain was contributed to the construction of the development theory of sociability using the results of the cohort studies and this development also suggested many hypotheses. For example, “the good reputation from others” as a cause of altruistic behavior was proved to activate a reward system which was similar to a money reward.^19^ This “praise” was expected to induce the sociability.

As the progression of the study, a lot of measurement methods were developed and the reliability and validity of these were also examined. For example, the observation item for 5 years old children which was used by physician was developed and this item was representative example to estimate the characteristic of the development process of the sociability using observation of children’s behavior. Moreover, this item was more useful to identify the problem of the development than other methods such as development of “the relation index”, development (a behavior measurement group) of the measurement technique to obtain evaluation index of the society ability from the image analysis of the play type balance game scene that used a motion capture system, and a general development evaluation.

Next, there were some findings from the preschool cohort. 1) The overprotection and excessive interference of parents for their children at 7 years old significantly affected the prosocial behavior of children at 8 years old. 2) Through the neurobehavioral examinations, 10 children were diagnosed as developmental disorders at 8 years old. The examination at five years old predicted at high probability the children diagnosed as attention deficit/hyperactivity disorders and pervasive developmental disorders at the 8 years of age. 3) In neuroethics, the protocol for incidental findings was established.

## LIMITATIONS

The present study had several limitations. First, the sample size was too small to analyze in detail, because these cohorts were set as a pilot study for a large scale cohort study. And participants were not by the random sampling. Secondly, because there were a few measurements for evaluation of child sociability, we had to develop the new methods. This might make it difficult to compare the results of the present study with previous studies.

## DISCUSSIONS

This study was interdisciplinary and consisted of “development cohort study”, “neuroscience” and “neuroethics”. Moreover, this study was conducted with “individual physician observation” in the entire period. While there were various cohort studies about development all over the world,^8^^,^^20^^–^^22^ these studies did not conduct under this study design. Therefore, the originality of our study is extremely higher than other studies.

This study was located as a development cohort study, neuroscience and each side of the neuroethics because there was no interdisciplinary study before this study.

The characteristic of this study as “the development cohort study” is summarized in following three points. First, the finding of this study was the untrodden region by the regional bridging positively. Second, this study also secured observation precision by physician observation, and realized practical use to medical examinations. Third, this study accomplished to use an established index which was internationally validated, and enabled to compare the results with the previous cohort studies.

Next, regarding the analysis, this study was successful to determine the effect of development factor which was characterized as the universal effect factor and Japanese current situation which was surrounding children. Development of recent non invasive cerebral function imaging technology enabled to clarify that nerves base of the higher brain function was able to include sociability. Moreover, functional analysis of autopsy technique in the brains study also enabled to advance the study about “the neuroscience.”

This study was one of trial to enable to combine a neuroscientific study with cohort vertical section observation and to analyze the acquisition process when the sociability in the development period was substantially normal. We “praised” it, and resulted by this “cohort study supported by a finding of the neuroscience” includes (social approval). From the results of functional MRI in this study, “the good reputation from others” as a cause of altruistic behavior was proved to activate a reward system similar to a money reward. It was actually shown that we “praised” it, and to have an influence as the reward on a development of the sociability by cohort analysis of this study. This shows that it is effective to combine a cohort study with neuroscience organically in a construction and the inspection of the development theory of the sociability.

Regarding a neuroethics, there were a lot of problems of the neuroethics for children, and was the neuroethics in the cohort study. It was still unclear what kind of the framework of neuroethics as a developmental cohort study. We also point out that scientists and research institutions of neuroscience should take more important role. Neuroscientists who study children may be responsible for children’s life in another way from the parents, schoolteachers, medical doctors. Basic scientists today should arrange their knowledge to more suitable form for the public, and should be open the both-way channel for communication with the public. We need further discussion how to realize interaction, which is more fruitful between local community and basic science community.

Thus, a lot of important and valuable findings for the future large scale cohort study were obtained because of the systematic conduct of the study. We have almost prepared a long-team and large scale cohort study on development sociability in childhood.

## APPENDIX

### Japan Children’s Study Group

Chairman: Zentaro Yamagata (Department of Health Sciences, School of Medicine, University of Yamanashi), Hideaki Koizumi (Advanced Research Laboratory, Hitachi, Ltd.).

Participating Researchers: Yoko Anji, Yuka Shiotani, Mizue Iwasaki, Aya Kutsuki, Misa Kuroki, Naho Ichikawa, Tomoyo Morita, Haruka Koike, Yusuke Morito, Shunyue Cheng, Hiraku Ishida, Hisakazu Yanaka, Daisuke Tanaka, Kumiko Namba, Tamami Fukushi, Hiroshi Toyoda, Shihoko Kimura-Ohba, Akiko Sawada (Research Institute of Science and Technology for Society, Japan Science and Technology Agency), Kevin K. F. Wong (Department of Anesthesia and Critical Care, Massachusetts General Hospital), Yoichi Sakakihara (Department of Child Care and Education, Ochanomizu University), Hideo Kawaguchi (Advanced Research Laboratory, Hitachi, Ltd.), Toyojiro Matsuishi (Department of Pediatrics and Child Health, Kurume University), Shunya Sogon (The Graduate Divisiton of the faculty of Human Relations, Kyoto Koka Women’s University), Kiyotaka Tomiwa, Tomonari Awaya, Sigeyuki Matuzawa (Graduate School of Medicine, Kyoto University), Shoji Itakura (Graduate School of Letters, Kyoto University), Masako Okada (Koka City Educational Research Center), Yoshihiro Komada (Department of Pediatric and Developmental Science, Mie University Graduate School of Medicine Institute of Molecular and Experimental Medicine), Hatsumi Yamamoto, Noriko Yamakawa, Motoki Bonno (Clinical Research Institute, Mie-chuo Medical Center, National Hospital Organization), Mariko Y. Momoi, Takanori Yamagata, Hirosato Shiokawa (Department of Pediatrics., Jichi Medical University), Norihiro Sadato, Daisuke N Saito (National Institute for Physiological Sciences, National Institutes of Natural Sciences), Hitoshi Uchiyama (Matsue Co-medical College), Tadahiko Maeda, Tohru Ozaki (The Institute of Statistical Mathematics, Research Organization of Information and Systems), Tamiko Ogura (Graduate School of Humanities, Kobe University), Hiroko Ikeda (National Epilepsy Center Shizuoka Institute of Epilepsy and Neurological Disorder), Koichi Negayama (Graduate School of Human Sciences, Waseda University), Kayako Nakagawa (Graduate School of Engineering, Osaka University), Kanehisa Morimoto (Graduate School of Medicine, Osaka University), Tokie Anme (Graduate School of Comprehensive Human Sciences, University of Tsukuba), Katsutoshi Kobayashi (Center for Education and Society, Tottori University), Tatsuya Koeda, Toshitaka Tamaru, Ayumi Seki, Shinako Terakawa, Ariko Takeuchi (Faculty of Regional Sciences, Tottori University), Yukuo Konishi (Department of Infants’ Brain & Cognitive Development, Tokyo Women’s Medical University), Osamu Sakura (Interfaculty Initiative in Information Studies, The University of Tokyo), Masatoshi Kawai (Institute for Education, Mukogawa Women’s University), Sonoko Egami (Hokkaido University of Education), Takahiro Hoshino (Graduate School of Economics, Nagoya University), Yuko Yato (College of Letters, Ritsumeikan University).
